# 

**Published:** 1898-04

**Authors:** 


### EDITORIAL.

---

The office of THE JOURNAL is 311 and 312 Fitten Building.

Address all communications, and make all remittances payable to THE ATLANTA MEDICAL AND SURGICAL JOURNAL, Atlanta, Ga.

Reprints of articles will be furnished, when desired, at a small cost. Requests for the same should always be made *on the manuscript.*

---

### THE MEDICAL ASSOCIATION OF GEORGIA.

The forty-ninth annual session of the State Association will be held at Cumberland Island April 20th, 21st and 22d. It is hoped that all who can do so will make it a point to be present. Following is a partial list of papers that have been promised:

1. Treatment of Typhoid Fever. Wm. J. Cox, M.D., Barnesville.

2. A Report of Surgical Cases. Hunter P. Cooper, M.D., Atlanta.

3. Malarial Asthenopia. J. Lawton Hiers, M.D., Savannah.

4. Diphtheria Antitoxin with Report of Cases. Henry R. Slack, M.D., LaGrange.

5. Solid Tumors of the Ovary. Virgil O. Hardon, M.D., Atlanta.

6. Puerperal Eclampsia and some Probable Causes of It. J. I. Griffeth, M.D., Danielsville.

7. Hysteria. A. A. Davidson, M.D., Augusta.

8. Femoral Hernia, with Report of Cases. Floyd W. McRae, M.D., Atlanta.

9. Primary Sarcoma of the Orbit, with Cases. Alex. W. Stirling, M.D., Atlanta.

10. Mushrooms, a Food and a Poison. W. H. Elliott, M.D., Savannah.

11. The Indication for and Antiseptic Technique of Uterine Drainage after Labor and Abortion. R. R. Kime, M.D., Atlanta.

12. A Rare Form of Bone Atrophy following an Ununited Fracture, as seen by the X-Ray. Eugene R. Corson, M.D., Savannah.

13. Surgical Treatment of Hemorrhoids. Wm. S. Goldsmith, M.D., Atlanta.

14. A few Cases from my Note-Book. J. G. Hopkins, M.D., Thomasville.

15. The Possibilities of a Perfect Aseptic Surgical Technique. E. C. Davis, M.D., Atlanta.

16. Diphtheria, its Present Status and Treatment. B. C. Powell, M.D., Villa Rica.

17. Shall Georgia have a Medical Law Pertaining to Marriage Contracts? J. Chestnut King, M.D., Newnan.

18. Tonsillotomy, When and How to Make It. J. M. Crawford, M.D., Atlanta.

19. A Georgia Incubator. W. P. Williams, M.D., Blackshear.

20. Some Remarks on (*a*) Appendicitis, (*b*) Hernia. W. F. Westmoreland, M.D., Atlanta.

21. Chronic Diarrhea as an Entity, with Report of Cases. T. M. Greenwood, M.D., Mineral Bluff.

22. A Few Remarks on the Treatment of Post-Nasal Adenitis. M. F. Carson, M.D., Griffin.

23. The Physician. J. S. Wimberly, M.D., Sunlight.

24. The Endoscopic Treatment of Chronic Urethritis, with Report of Cases. W. L. Champion, M.D., Atlanta.

25. Antitoxin in the Treatment of Diphtheria. B. L. Bridges, M.D., Ellaville.

26. Adenoids in the Pharyngeal Vault and Their Influence upon the Ear. A. W. Calhoun, M.D., Atlanta.

27. The Deadbeat *vs.* The Doctor. W. J. Rowe, M.D., Buford.

28. Report of a Case of Poliomyelitis. W. L. Patten, M.D., Milltown.

29. Some Pathological Investigations. Chas. M. Blackford, M.D., Augusta.

30. (Title to be furnished.) S. Latimer Phillips, M.D., Savannah.

31. Alcoholic Irrigation in Puerperal Sepsis. Geo. H. Noble, M.D., Atlanta.

32. The Importance of Careful Chemical Analysis in Gastric Disorder. Wm. Clifton Lyle, M.D., Augusta.

The Cumberland Island Hotel will be headquarters for members of the Association, and the place of meeting will be the Georgia Teachers' Auditorium adjacent to the hotel.

The committee in charge of transportation will secure low railroad rates, perhaps lower than have heretofore been granted the Association. Several pleasant excursions from Brunswick have been arranged for the social entertainment of the visiting members, and every effort will be made to make this meeting profitable and enjoyable, in both scientific and social senses. The Committee of Arrangements wants all to come.

On Tuesday the 19th, the day before the assembling of the Association, the eighth annual meeting of the Central of Georgia Railway Surgeons Association will be held in the parlors of the Cumberland Island Hotel. An interesting program has been prepared and a pleasant meeting is expected.

